# Long-Term Management of Adolescent Idiopathic Scoliosis in Adulthood: Clinical Challenges and Progress During the Critical Period of Pregnancy

**DOI:** 10.3390/healthcare14070856

**Published:** 2026-03-27

**Authors:** Zhaomeng Hou, Jiaojiao Wu, Chaoqun Chen, Hua Lu, Jiali Yang, Tingliang Han

**Affiliations:** 1 Department of Orthopedics and Traumatology, Yancheng TCM Hospital Affiliated to Nanjing University of Chinese Medicine, Yancheng 224000, China; 2 Department of Pharmacology, School of Basic Medical Science, Nanjing Medical University, Nanjing 211166, China

**Keywords:** idiopathic scoliosis, adult patients, long-term management, pregnancy, spine-pelvic biomechanics, pain management

## Abstract

Adolescent idiopathic scoliosis (AIS) is the most common three-dimensional spinal misalignment, with its impact often extending into adulthood and presenting a range of long-term health challenges. This review focuses on the long-term management strategies for adult patients with AIS and explores the unique physiological event of pregnancy and its influence on disease progression, clinical symptoms, and treatment decisions. We systematically analyze the natural history of adult AIS, the evolution of pain and functional impairment, and radiographic progression patterns. Special emphasis is placed on biomechanical changes of the spine and pelvis during pregnancy and postpartum, pain management approaches, delivery mode selection, and potential effects on offspring. By integrating current evidence and recent research findings, this review synthesizes key insights: the progression of AIS in adulthood is typically slow but can be exacerbated by factors such as significant curve magnitude at skeletal maturity; pregnancy does not consistently accelerate curve progression but may intensify pain and functional limitations, necessitating tailored multidisciplinary management; and evidence supports that most women with AIS can undergo vaginal delivery without increased obstetric risk, while anesthetic and analgesic planning requires careful consideration of spinal anatomy. This review aims to provide evidence-based guidance and clinical recommendations for comprehensive lifelong health management of this special patient population.

## 1. Introduction

Adolescent idiopathic scoliosis (AIS) is a complex three-dimensional structural spine disorder of unknown etiology, typically diagnosed during adolescence [1]. While much attention has been devoted to the diagnosis and management of AIS during the growth period, it is increasingly recognized that the implications of AIS extend well into adulthood and even old age. The majority of individuals diagnosed with AIS in adolescence continue to carry their spinal curvature into adulthood, where they face a unique set of long-term health challenges. These include progressive worsening of spinal asymmetry, degenerative changes in the spine, chronic pain, compromised cardiopulmonary function, and significant psychosocial burdens. Such multifaceted issues create distinct clinical management challenges that differ substantially from those encountered in the adolescent population. The natural history of AIS in adults is characterized by a variable progression of spinal curvature, often accompanied by compensatory postural adaptations and degenerative spinal changes that can exacerbate symptoms and functional limitations. For instance, a retrospective 10-year analysis of unoperated adult patients with AIS demonstrated that while most adults function well, a subset experiences increased disability correlated with age, body mass index, and curve magnitude [2]. This underscores the importance of long-term surveillance and individualized management strategies tailored to the evolving clinical status of adult patients with AIS.

Non-operative management remains a cornerstone of treatment for adults with structural spine disorders, including AIS, especially in patients without neurological deficits or severe functional impairment. Although rigid bracing is well established in adolescent populations, its role in adults has been less clearly defined. Recent studies have begun to elucidate the benefits of conservative interventions such as scoliosis support orthoses (SSO) and physiotherapy. For example, a study evaluating the impact of SSO bracing in symptomatic adults with structural spine disorder patients reported significant improvements in pain, gait parameters, and dynamic balance after both short-term and extended use of the orthosis [3]. Similarly, Schroth-based physiotherapy combined with part-time bracing has shown promise in reversing postural and functional decline in complex post-fusion adult AIS cases, leading to meaningful improvements in spinal alignment, pulmonary function, and quality of life measures [4]. This finding highlights the potential role of conservative modalities in the post-surgical management of adult patients with AIS. Furthermore, conservative approaches may also mitigate symptoms and improve function in patients who are not candidates for surgery or who decline operative intervention.

Surgical management in adults with AIS is often considered when there is significant curve progression, pain, or functional impairment. However, surgery in this population carries increased risks due to factors such as comorbidities, spinal rigidity, and the presence of degenerative changes. Moreover, postoperative outcomes can be influenced by preoperative pain trajectories and psychosocial factors. A study investigating acute postoperative pain trajectories in adolescents undergoing spinal fusion found that preoperative pain catastrophizing and analgesic use predicted pain patterns post-surgery, which in turn correlated with long-term pain severity and functional disability [5]. Although this study focused on adolescents, the insights regarding pain trajectory and long-term outcomes are relevant to adult patients with AIS undergoing surgery, emphasizing the need for comprehensive preoperative assessment and tailored perioperative pain management to optimize recovery and quality of life.

Pregnancy introduces significant physiological challenges for women with AIS, adding complexity to their long-term management. Hormonal changes, substantial weight gain, and biomechanical adaptations in the spine and pelvis during pregnancy may influence scoliosis progression and exacerbate symptoms. A retrospective study of 163 AIS patients with 263 pregnancies found that pregnancy worsened back pain in most cases and posed challenges for administering neuraxial anesthesia during delivery [6]. Notably, some patients showed measurable progression of spinal curvature after childbirth, suggesting that pregnancy can affect the natural history of AIS. Anesthesia difficulties, including a 6.1% incidence of challenges with neuraxial anesthesia, further complicate obstetric care for these patients. These findings highlight the need for multidisciplinary collaboration among spine specialists, obstetricians, anesthesiologists, and physiotherapists to develop comprehensive care pathways tailored to the unique needs of pregnant women with AIS.

Despite the growing recognition of the long-term implications of AIS in adulthood and the specific challenges posed by pregnancy, current clinical guidelines remain fragmented and insufficiently detailed. There is a pressing need for systematic reviews and evidence synthesis to consolidate knowledge on the longitudinal management of adult patients with AIS, with particular attention to pregnancy-related issues. This systematic review aims to fill this knowledge gap by providing a comprehensive overview of the current state of research. Specifically, it seeks to synthesize evidence on the effectiveness and outcomes of both surgical and non-surgical treatments in the long-term management of adult AIS, with a focus on patient-reported outcomes, radiographic progression, and pregnancy-related complications. The review thereby aims to support the development of standardized protocols and multidisciplinary models to optimize clinical outcomes and quality of life for this patient population.

## 2. Methodology

To ensure a comprehensive and systematic evidence synthesis, a structured literature search was conducted. The search strategy was designed to identify all relevant studies pertaining to the long-term management of adult AIS and pregnancy-related considerations. Electronic databases, including PubMed/MEDLINE, Embase, and the Cochrane Library, were systematically searched from inception to February 2026. The search utilized a combination of Medical Subject Headings (MeSH) terms and keywords related to “adolescent idiopathic scoliosis,” “adult,” “long-term outcomes,” “pregnancy,” “management,” and “quality of life.” The reference lists of included articles and relevant review papers were also manually screened to identify additional studies.

Studies were selected based on predefined inclusion and exclusion criteria. Inclusion criteria encompassed: (1) original research articles, systematic reviews, and meta-analyses; (2) study populations of adults (aged ≥ 18 years) with a diagnosis of AIS; (3) focus on long-term health outcomes, management strategies, or pregnancy-related issues; and (4) publication in English. Exclusion criteria included: (1) studies focused exclusively on pediatric or adolescent populations; (2) case reports, editorials, and conference abstracts with insufficient methodological detail; and (3) studies not directly relevant to the clinical management or pregnancy outcomes in AIS.

The study selection process involved two independent reviewers. Initially, titles and abstracts of all retrieved records were screened against the eligibility criteria. Subsequently, the full texts of potentially relevant articles were assessed for final inclusion. Any discrepancies between reviewers were resolved through discussion or consultation with a third reviewer. Data from the included studies were then extracted and synthesized narratively to address the review’s objectives.

## 3. Natural History and Long-Term Prognosis of Adult AIS

### 3.1. Progression Patterns of Cobb Angle in Adulthood

The progression of scoliosis in adulthood, particularly in idiopathic cases, is influenced by multiple factors including the initial Cobb angle magnitude, vertebral apex location, and skeletal maturity as indicated by the Risser sign. Studies consistently demonstrate that larger curves at skeletal maturity are more prone to progression during adulthood. Specifically, thoracic curves exceeding 50 degrees and thoracolumbar or lumbar curves greater than 30 degrees are associated with a higher risk of progression, with an average annual increase in Cobb angle ranging from 0.5 to 1 degree [7]. This slow but steady progression underscores the importance of long-term monitoring even after skeletal maturity is reached.

Lumbar scoliosis, especially when accompanied by vertebral rotation and intervertebral disc degeneration, exhibits a more aggressive progression pattern compared to thoracic curves. The presence of rotational subluxation and disc degeneration not only accelerates curve progression but also correlates more strongly with symptomatic lumbar pain [8]. This association highlights the clinical relevance of lumbar curve characteristics in predicting both radiographic and symptomatic outcomes.

Postmenopausal women represent a subgroup at increased risk for scoliosis progression due to accelerated bone loss and osteoporosis. The decline in bone mineral density compromises vertebral structural integrity, potentially facilitating curve worsening in patients with pre-existing deformities [9]. This phenomenon suggests that hormonal and metabolic factors contribute to the natural history of adult scoliosis, necessitating tailored management strategies in this population.

Radiographic studies comparing AIS and adult idiopathic lumbar scoliosis reveal that thoracolumbar/lumbar curves tend to progress with increased kyphosis and subsequent sagittal imbalance as patients age [10]. Lumbar lordosis and pelvic tilt deteriorate linearly with age, and sagittal balance is maintained only until approximately 50 years of age, after which compensatory mechanisms fail, leading to clinical deterioration. These findings emphasize the dynamic nature of spinal alignment changes over the adult lifespan and the importance of sagittal plane assessment in adult scoliosis.

The heterogeneity of adult idiopathic scoliosis has led to the development of classification systems distinguishing young adult idiopathic scoliosis (19–30 years) from older adult idiopathic scoliosis (>30 years), with further subdivisions based on curve flexibility and stiffness [11]. This classification reflects the progressive stiffening and degenerative changes that influence curve behavior and surgical planning. Notably, older adults tend to have stiffer curves with more degenerative features, which may affect the rate and pattern of progression.

Nonoperative management, including bracing and physiotherapy, has shown some efficacy in controlling curve progression and alleviating symptoms in adults with scoliosis, although evidence remains limited and of low quality [12,13]. Bracing may modestly reduce pain and improve function, but its impact on Cobb angle progression is variable. Conservative treatments are particularly relevant for patients with mild to moderate curves or those who are not surgical candidates.

Surgical intervention in adults is often considered when curves exceed 45–50 degrees or when symptomatic progression occurs. However, surgery in adults carries higher morbidity and complexity compared to adolescents, partly due to curve stiffness and degenerative changes [14]. Postoperative outcomes and complication rates differ between adolescent and adult patients, with adults requiring longer operative times, more extensive fusion levels, and experiencing greater blood loss.

Emerging technologies such as machine learning are being explored to predict curve progression and optimize management strategies, although current models require further validation [15]. Additionally, novel surgical techniques, including posterior-only approaches and multilevel posterolateral convex disc releases, have been developed to address the challenges of rigid adult curves [16,17].

In summary, the progression of Cobb angle in adult idiopathic scoliosis is a multifactorial process influenced by initial curve magnitude, curve location, skeletal maturity, degenerative changes, and systemic factors such as osteoporosis. Thoracic curves over 50 degrees and lumbar curves over 30 degrees at skeletal maturity are at higher risk for progression, with an average annual increase of 0.5 to 1 degree. Lumbar curves with rotational subluxation and disc degeneration progress more rapidly and are more symptomatic. Postmenopausal bone loss further exacerbates progression risk. Long-term monitoring and individualized management, incorporating both conservative and surgical options, are essential to optimize outcomes in this population.

### 3.2. Degenerative Changes and Evolution of Clinical Symptoms

Adult patients with AIS exhibit a markedly higher incidence and earlier onset of spinal degenerative changes compared to the general population. These degenerative alterations primarily involve intervertebral disc degeneration, facet joint arthritis, and spinal canal stenosis. Radiographic and magnetic resonance imaging (MRI) studies have demonstrated that, even after surgical intervention such as selective thoracic fusion (STF), degenerative changes persist or progress in the lumbar spine, particularly in the unfused segments. For instance, a long-term follow-up study of AIS patients who underwent STF revealed significant disc height reduction in lumbar segments, although the degree of radiographic degeneration did not differ significantly from matched controls, suggesting an accelerated but not necessarily pathological degenerative process in these patients [18]. Furthermore, facet joint degeneration and paraspinal muscle fatty infiltration have been observed postoperatively, with the extent of degeneration influenced by the lowest instrumented vertebra (LIV). Patients with more distal LIVs (L3 or below) showed greater facet joint and paraspinal muscle degeneration compared to those with proximal LIVs (L2 or above), although these radiological changes did not correlate strongly with clinical symptoms at five years post-surgery [19].

Chronic low back and lumbar pain represent the most prevalent clinical manifestations in adult patients with AIS, with severity variably associated with the magnitude of the scoliotic curve, degree of degenerative changes, trunk imbalance, and pelvic tilt. However, the relationship between these factors and pain intensity is complex and not strictly linear. Clinical outcome studies indicate that patients with more severe preoperative symptoms tend to experience greater postoperative improvement, yet they are less likely to achieve asymptomatic status, highlighting the chronic and multifactorial nature of pain in this population [20]. The paravertebral muscles, especially the multifidus, exhibit asymmetric degeneration with greater fatty infiltration on the concave side of the curve, which is exacerbated in patients with sagittal imbalance characterized by pelvic incidence-lumbar lordosis mismatch. This muscular degeneration correlates positively with pain intensity, symptom duration, and disability indices, underscoring its role in symptom evolution [21].

Neurological symptoms in adult patients with AIS, such as radiculopathy and neurogenic claudication, may potentially arise from mechanical factors related to the scoliotic asymmetry and associated degenerative changes. Nerve root compression may result from foraminal stenosis on the concave side due to disc space narrowing and osteophyte formation, or from nerve root traction on the convex side caused by lateral listhesis and asymmetry-induced tension. A retrospective study assessing transforaminal epidural steroid injection efficacy in degenerative lumbar scoliosis patients with stenosis found that injections on the convex side of the primary lumbar curve provided superior symptomatic relief for radicular pain, particularly at the L5-S1 level, compared to concave side injections. This suggests that nerve root irritation mechanisms differ by curve side and that convex side pathology may be more amenable to targeted interventions [22]. Conversely, spinal cord compression symptoms are relatively rare in untreated adult AIS unless complicated by severe kyphotic asymmetry or degenerative spondylolisthesis, which can cause central canal stenosis and myelopathy [23].

In addition to degenerative and mechanical factors, complex cases involving neurological compromise have been reported. For instance, while Charcot arthropathy secondary to adult degenerative scoliosis combined with syringomyelia is considered rare, its occurrence illustrates the potential for severe joint destruction and neurological sequelae in advanced disease [24]. Moreover, degenerative changes in the lumbar spine can contribute to vascular complications. A notable example is the bony May-Thurner syndrome caused by lumbar osteophytes compressing the left common iliac vein. This condition is also uncommon but may coexist with degenerative scoliosis, where its presence can significantly exacerbate clinical symptoms [25].

Surgical interventions aimed at correcting adult scoliosis and its degenerative sequelae, including lateral lumbar interbody fusion (LLIF), oblique lumbar interbody fusion (OLIF), and spinopelvic fixation with S2-alar-iliac screws, have demonstrated efficacy in improving spinal alignment and reducing pain. However, these procedures also influence the progression of degenerative changes and clinical symptoms. For example, LLIF has been shown to significantly reduce Cobb angles and improve functional outcomes with relatively low morbidity, while OLIF offers advantages in indirect decompression and faster recovery but carries risks such as cage subsidence, especially in stand-alone constructs [26,27]. Spinopelvic fixation can alter pelvic parameters, including pelvic incidence, challenging the traditional view of fixed pelvic morphology and impacting sagittal balance, which is closely linked to symptom severity [28].

In summary, adult patients with AIS experience accelerated and multifactorial degenerative changes that contribute to a spectrum of clinical symptoms ranging from chronic axial pain to radiculopathy. The interplay between structural asymmetry, degenerative progression, muscular degeneration, and neurological involvement shapes the clinical course. While surgical correction can ameliorate symptoms and improve alignment, careful consideration of degenerative status and biomechanical factors is essential for optimizing long-term outcomes. Continued longitudinal studies integrating radiographic, clinical, and functional data are necessary to elucidate the natural history and refine management strategies for this complex patient population.

## 4. Non-Surgical Management and Long-Term Monitoring of Adult Patients with AIS

### 4.1. Pain Management and Rehabilitation Strategies

Effective pain management and rehabilitation are central to the long-term care of adult patients with idiopathic scoliosis, aiming to alleviate symptoms, improve function, and enhance quality of life. Multimodal pain management constitutes the cornerstone of treatment, integrating pharmacological, physical, interventional, and psychological approaches. Nonsteroidal anti-inflammatory drugs remain a first-line pharmacologic option for controlling musculoskeletal pain associated with scoliosis, providing symptomatic relief with a favorable safety profile when used appropriately. Complementing medication, physical therapy plays a pivotal role, focusing on core muscle strengthening, postural training, and flexibility exercises to reduce spinal load and improve biomechanical stability. Specific modalities such as sensorimotor training combined with core strength training have been proposed to target low back pain in adult idiopathic scoliosis, with ongoing randomized controlled trials designed to evaluate their efficacy in reducing pain and enhancing functional status [29]. Selective nerve blocks may be employed in cases of radicular pain or nerve root irritation, offering targeted analgesia and potentially reducing reliance on systemic medications. Psychological interventions addressing pain coping strategies and mental health are also integral, given the documented association between scoliosis-related pain and psychological distress.

Among physical therapy techniques, scoliosis-specific exercises, particularly the Schroth method, have demonstrated efficacy in improving pain, respiratory function, and self-image in adult patients. Schroth exercises emphasize three-dimensional corrective movements, rotational angular breathing, and postural awareness, aiming to optimize spinal alignment and muscular balance. A randomized controlled trial comparing Schroth combined with core training versus core training alone in AIS patients postoperatively revealed superior improvements in pelvic balance, trunk extensor endurance, and self-image in the Schroth group, although no significant differences were observed in Cobb angle correction or pain relief between groups [30]. Furthermore, a unique 35-year case study of an adult AIS patient post-hardware removal demonstrated that a prolonged Schroth-based physiotherapy and bracing program could substantially reduce thoracic kyphosis and coronal curve magnitude, improve pulmonary function by 33%, and eliminate disability as measured by the Oswestry Disability Index, underscoring the potential of this conservative approach in complex post-fusion scoliosis [4]. However, it is important to note that while Schroth exercises can improve symptoms and function, their ability to halt or reverse curve progression in adults remains limited.

Regular engagement in low-impact aerobic exercise, such as swimming and walking, is recommended to maintain overall health, control body weight, and reduce mechanical stress on the spine. These activities promote cardiovascular fitness without imposing excessive axial loading, which is particularly beneficial in adult scoliosis patients who may have compromised spinal biomechanics. Additionally, dynamic neuromuscular stabilization combined with core stability exercises has shown promise in improving lumbopelvic stability in individuals with idiopathic scoliosis, which may translate into better postural control and reduced pain [31]. Rehabilitation programs incorporating sensorimotor training and core strengthening are being investigated to optimize pain management and functional outcomes, reflecting a trend toward individualized, multimodal rehabilitation strategies.

Bracing remains a conservative treatment option in adult scoliosis, particularly for those with progressive curves or pain. A systematic review of spinal brace and orthosis use in adults with scoliosis reported modest to significant reductions in pain and functional improvements, although evidence quality was low and primarily focused on female patients with thoracolumbar and lumbar curves [12]. Bracing prescriptions varied widely, and compliance was mixed, highlighting the need for further high-quality prospective studies to clarify efficacy and optimize protocols. In addition to conventional bracing, innovative rehabilitation technologies such as brain–computer interface-assisted climbing wall training have been explored in AIS, demonstrating significant reductions in Cobb angle, improved lumbar range of motion, and enhanced quality of life, suggesting potential future applications in adult populations [32].

Postoperative pain management and rehabilitation are critical for optimizing recovery in scoliosis patients undergoing surgery. The implementation of rapid recovery pathways with multimodal analgesia and early mobilization has been shown to reduce pain scores, shorten hospital stays, and accelerate physical therapy clearance in AIS surgery, which may inform strategies for adult patients as well [33]. Moreover, telerehabilitation programs initiated six months post-surgery have demonstrated significant improvements in trunk muscle endurance, flexibility, pain intensity, and quality of life, emphasizing the value of structured, supervised rehabilitation even in the outpatient setting [34].

Traditional Chinese medicine modalities, including acupuncture and massage, have also been investigated as adjuncts to rehabilitation following scoliosis surgery. A retrospective study involving patients treated with 3D printing orthopedic surgery found that postoperative acupuncture and massage significantly improved vertebral rotation angles, Cobb angles, pain scores, and functional disability indices compared to conventional rehabilitation alone, suggesting a beneficial role in enhancing spinal function and pain control [35].

Pain in idiopathic scoliosis is multifactorial, often associated with muscular imbalances, postural instability, and psychological factors. Studies have highlighted the relationship between pain severity and poor sleep quality in scoliosis patients, underscoring the importance of comprehensive pain management that addresses both physical and psychosocial dimensions [36]. Muscle fatigue, particularly of the thoracic erector spinae on the convex side of the curve, may exacerbate pain and impair neuromuscular control, indicating that endurance training targeting these muscles could be a valuable component of rehabilitation [37].

In summary, pain management and rehabilitation in adult idiopathic scoliosis require a multimodal, individualized approach. Pharmacologic treatments such as nonsteroidal anti-inflammatory drugs provide symptomatic relief, while targeted physical therapies—including Schroth exercises, core strengthening, sensorimotor training, and aerobic conditioning—address biomechanical and functional deficits. Bracing may offer additional benefits in selected patients. Postoperative rehabilitation protocols incorporating multimodal analgesia, early mobilization, and telerehabilitation enhance recovery and long-term outcomes. Adjunctive therapies such as traditional Chinese medicine and emerging technologies hold promise for further improving pain and function. Ongoing research and high-quality clinical trials are essential to refine these strategies and establish standardized guidelines for optimal long-term management of adult idiopathic scoliosis patients.

### 4.2. Regular Imaging and Functional Status Assessment

Long-term management of adult patients with AIS necessitates systematic and periodic evaluation of spinal asymmetry progression and functional status to guide clinical decision-making and optimize outcomes. Establishing a comprehensive longitudinal follow-up registry for adult patients with AIS is recommended, with clinical assessments and standing full-spine radiographs performed at regular intervals, typically every 2 to 5 years. This approach enables monitoring of key parameters such as Cobb angle progression, trunk balance, and degenerative changes within the spinal column. Quantitative imaging modalities, including low-dose stereoradiography (e.g., EOS imaging), provide three-dimensional assessments of spinal alignment with reduced radiation exposure, facilitating repeated evaluations over time without undue risk [38,39]. Digital measurement techniques allow precise quantification of coronal and sagittal anatomic parameters, which have been shown to correlate with scoliosis severity and progression risk [40]. Additionally, three-dimensional ultrasound imaging has demonstrated high intra- and inter-evaluator reliability for coronal plane vertebral displacement measurements, offering a radiation-free alternative for monitoring [41]. For patients exhibiting new or progressive neurological symptoms or severe pain, timely advanced imaging with MRI or computed tomography (CT) is essential to evaluate the spinal canal, nerve roots, and intervertebral discs. MRI is particularly valuable for detecting occult neural axis abnormalities, such as Chiari malformation or syringomyelia, which may influence surgical planning and prognosis even in neurologically intact patients [42,43,44]. MRI also facilitates assessment of paraspinal muscle imbalance, which correlates with curve magnitude and may reflect compensatory adaptations [45]. In cases of severe thoracic curves exceeding 70–80 degrees or when respiratory symptoms are present, pulmonary function testing is critical to evaluate the impact of asymmetry on respiratory mechanics. Studies have shown that pulmonary function can improve following corrective interventions, and baseline assessment aids in risk stratification and management planning [23,46]. Furthermore, functional status and quality of life should be regularly assessed using validated patient-reported outcome measures such as the Scoliosis Research Society questionnaires (SRS-22r or SRS-30), Oswestry Disability Index (ODI), and EuroQol-5D, which provide insights into pain, self-image, mental health, and satisfaction with treatment [47,48]. These assessments are integral to holistic care, enabling tailored interventions including physiotherapy, bracing, or surgical options. In summary, a structured protocol involving periodic clinical and radiographic evaluations, supplemented by advanced imaging and functional testing as indicated, is paramount for effective long-term surveillance and management of adult patients with AIS. This strategy facilitates early detection of progression, neurological compromise, or pulmonary impairment, thereby guiding timely and individualized therapeutic decisions.

## 5. Indications and Challenges of Surgical Treatment for Adult Patients with AIS

### 5.1. Indications and Decision-Making for Delayed Surgery

In adult patients with idiopathic scoliosis, the decision to proceed with delayed surgical intervention is multifactorial and hinges on a constellation of clinical and radiographic indicators. The primary surgical indications in adulthood include progressive and worsening pain unresponsive to conservative management, documented curve progression typically exceeding 2 degrees per year, increasing trunk imbalance that impairs standing and ambulation, and the presence of neurological deficits or signs of spinal cord compression. These criteria underscore the necessity of surgery not only to halt asymmetry progression but also to address functional impairment and neurological compromise. The complexity of surgical decision-making in adults is heightened compared to adolescents due to the increased risk profile associated with age, comorbidities, and diminished bone quality. Patient age and overall health status, including comorbid conditions and bone mineral density, must be carefully evaluated to balance surgical benefits against potential complications. Additionally, the severity of symptoms and the degree of curve rigidity influence the surgical approach and expected outcomes. Patient expectations and goals are integral to the decision process, requiring thorough counseling regarding the risks, benefits, and realistic postoperative outcomes. Studies have demonstrated that adults undergoing scoliosis surgery often face higher operative morbidity and longer fusion constructs compared to adolescent patients [49], necessitating a tailored approach that considers individual patient factors and asymmetry characteristics. Furthermore, the timing of surgery is critical; delaying intervention until after skeletal maturity or completion of athletic participation may be advisable in select cases to optimize functional outcomes. The decision-making process is further complicated by the bimodal nature of revision surgeries in adults who had adolescent scoliosis surgery, with indications such as implant malposition, adjacent segment degeneration, and pseudoarthrosis emerging years after the initial procedure [50]. This highlights the importance of long-term surveillance and individualized surgical planning in adult scoliosis patients. Emerging technologies, including deep learning models, are being developed to assist in surgical decision-making and outcome prediction, potentially enhancing personalized care. Overall, the indication for delayed surgery in adult idiopathic scoliosis requires a comprehensive assessment of clinical progression, radiographic parameters, patient health status, and personal expectations to optimize surgical timing and strategy, acknowledging the increased risks inherent in adult spinal asymmetry surgery [51,52].

### 5.2. Surgical Technique Characteristics and Complications

- Long-segment fusion with instrumentation is often required, sometimes extending to the pelvis to correct severe imbalance. Osteotomy techniques (such as SPO, PSO, VCR) are increasingly applied in managing rigid deformities.

Surgical management of adult idiopathic scoliosis frequently necessitates extensive spinal fusion spanning multiple vertebral levels to achieve adequate asymmetry correction and restore spinal balance. In cases of severe coronal and sagittal imbalance, fusion may need to be extended distally to the pelvis to provide a stable foundation and prevent distal junctional failure. The complexity of adult asymmetry surgery is compounded by the rigidity of curves, often necessitating osteotomy techniques to achieve sufficient correction. Posterior column osteotomy (SPO), pedicle subtraction osteotomy (PSO), and vertebral column resection (VCR) are commonly employed to address stiff deformities and restore sagittal alignment. These osteotomies allow for controlled correction of angular deformities by resecting posterior elements (SPO), wedge-shaped vertebral body segments (PSO), or entire vertebral segments (VCR), respectively. The choice of osteotomy depends on the severity and rigidity of the asymmetry, as well as the desired degree of correction. Recent advances have seen increased utilization of these techniques in adult scoliosis surgery to improve outcomes in patients with fixed deformities. For example, PSO is particularly effective in correcting sagittal imbalance by providing substantial lordotic correction at a single level, while VCR is reserved for the most severe and rigid deformities requiring three-dimensional correction. The application of these osteotomies, combined with long-segment instrumentation, facilitates restoration of spinal alignment but also increases surgical complexity and risk [53,54].

- Common complications include pseudarthrosis, instrumentation failure, proximal or distal junctional kyphosis, neurological injury, and deep infection, with incidence rates higher than in adolescent patients.

Adult idiopathic scoliosis surgery is associated with a higher complication rate compared to AIS surgery, reflecting the increased complexity of asymmetry, comorbidities, and surgical invasiveness. Pseudarthrosis, or nonunion at the fusion site, is a frequent complication that compromises construct stability and may necessitate revision surgery. Instrumentation failure, including screw loosening, rod breakage, or implant pull-out, is also prevalent, particularly in long-segment fusions extending to the pelvis. Junctional kyphosis, either proximal junctional kyphosis or distal junctional kyphosis, represents a significant postoperative challenge characterized by abnormal kyphotic angulation adjacent to the fused segments, potentially leading to pain, asymmetry progression, and neurological compromise. Neurological complications, including nerve root injury and spinal cord deficits, occur more commonly in adult asymmetry surgery due to the extensive surgical exposure and osteotomies performed. Deep surgical site infections are another serious concern, with rates higher than in younger populations, often necessitating prolonged antibiotic therapy and surgical debridement. The incidence of these complications is influenced by patient factors such as age, comorbidities, bone quality, and surgical factors including operative time and blood loss. For instance, adult patients undergoing scoliosis correction have demonstrated higher rates of pseudarthrosis and neurological complications compared to adolescents, with some studies reporting overall complication rates exceeding 20% [53,55,56,57].

- Osteoporosis is a critical factor affecting the stability of instrumentation; preoperative assessment and anti-osteoporotic treatment are essential.

Bone quality plays a pivotal role in the success of spinal instrumentation and fusion, particularly in adult scoliosis patients who often present with varying degrees of osteoporosis. Osteoporotic bone compromises screw purchase and fixation strength, increasing the risk of implant loosening, pull-out, and subsequent construct failure. This is especially relevant in long-segment fusions and pelvic fixation where biomechanical demands are high. Preoperative evaluation of bone mineral density is therefore crucial to identify patients at risk. Dual-energy X-ray absorptiometry scans are commonly used to assess osteoporosis severity. In patients with confirmed osteoporosis or osteopenia, preoperative optimization with anti-osteoporotic agents such as bisphosphonates, teriparatide, or denosumab may improve bone quality and enhance fusion rates. Additionally, intraoperative strategies such as cement augmentation of pedicle screws can be employed to improve fixation in osteoporotic vertebrae. The importance of addressing osteoporosis is underscored by studies demonstrating higher rates of instrumentation failure and pseudarthrosis in patients with poor bone quality [58]. Thus, a multidisciplinary approach involving endocrinologists and bone metabolism specialists is recommended to optimize bone health before and after surgery, aiming to reduce complications and improve surgical outcomes.

In summary, surgical correction of adult idiopathic scoliosis often requires extensive fusion with advanced osteotomy techniques to manage rigid deformities and restore spinal balance. However, these procedures carry a higher risk of complications compared to adolescent cases, including pseudarthrosis, instrumentation failure, junctional kyphosis, neurological injury, and infection. Osteoporosis significantly impacts the stability of instrumentation, necessitating thorough preoperative assessment and medical management to optimize bone quality and reduce postoperative complications. Careful patient selection, meticulous surgical technique, and comprehensive perioperative management are essential to improve long-term outcomes in this challenging patient population.

## 6. Impact of Pregnancy on Spine-Pelvic Biomechanics in AIS

### 6.1. Physiological Changes During Pregnancy and Spinal Load

Pregnancy induces profound physiological and biomechanical changes that significantly affect the spine and its load-bearing capacity. One of the hallmark hormonal changes during pregnancy is the elevated levels of relaxin and other pregnancy-related hormones, which promote ligamentous laxity to facilitate childbirth. This increased ligamentous laxity theoretically raises the risk of spinal instability, particularly in the lumbar region where the mechanical demands are greatest. However, the specific impact of these hormonal changes on the spines of patients with AIS, whether fused or unfused, remains controversial. Some studies suggest that spinal fusion constructs, especially those extending to lower lumbar vertebrae such as L3 or below, may predispose pregnant women to increased low back pain due to altered biomechanics and reduced spinal flexibility, yet the risk of curve progression during pregnancy appears low in fused AIS patients [59]. The ligamentous relaxation may exacerbate microinstability in unfused AIS spines, but definitive evidence is lacking, necessitating further longitudinal studies to clarify these effects.

In addition to hormonal influences, the mechanical burden on the spine increases substantially during pregnancy due to weight gain and the anterior shift of the center of gravity. The gravid uterus and increased maternal body mass cause a compensatory increase in lumbar lordosis and anterior pelvic tilt, which amplify the load on the lumbar vertebrae and intervertebral discs. This biomechanical adaptation, while necessary for maintaining balance, can exacerbate pre-existing spinal deformities such as lumbar scoliosis or pelvic obliquity, leading to intensified low back pain and functional impairment. The increased lumbar lordosis and pelvic tilt have been associated with sacroiliac joint laxity and altered gait mechanics, which further contribute to spinal discomfort and may predispose to degenerative changes over time [60]. Patients with pre-existing lumbar scoliosis or pelvic asymmetry may experience more pronounced symptoms due to these biomechanical stresses, highlighting the importance of individualized assessment and management during pregnancy.

Moreover, the enlarging uterus during pregnancy alters the function of the abdominal musculature, particularly the rectus abdominis and oblique muscles, which play a critical role in core stability and spinal support. The stretching and weakening of these muscles reduce the ability to stabilize the lumbar spine effectively, thereby increasing the compensatory load on the paraspinal musculature and spinal ligaments. This diminished core stability can exacerbate spinal deformities and contribute to the development or worsening of low back pain. The compromised abdominal muscle function also affects postural control and may lead to maladaptive spinal compensations, further increasing mechanical stress on the vertebral column [60]. These changes underscore the need for targeted physical therapy interventions focusing on core strengthening and postural training to mitigate spinal load and improve functional outcomes in pregnant women with scoliosis.

In summary, the interplay of hormonal-induced ligamentous laxity, increased mechanical load from weight gain and altered posture, and compromised core muscle function collectively heightens the biomechanical demands on the spine during pregnancy. While these changes are adaptive to support fetal growth and prepare for delivery, they pose challenges for women with pre-existing spinal deformities such as AIS. The current literature indicates that although curve progression during pregnancy is uncommon in fused AIS spines, the risk of low back pain and spinal discomfort is elevated, particularly in those with lower instrumented vertebrae or unfused curves [59]. The precise effects of pregnancy-related hormonal and biomechanical changes on spinal stability and long-term spinal health in this population remain incompletely understood, warranting further research. Multidisciplinary management involving obstetricians, spine specialists, and physical therapists is essential to optimize maternal musculoskeletal health and minimize pregnancy-related spinal complications.

### 6.2. Pelvic Adaptive Changes and Birth Canal Assessment

AIS frequently presents with pelvic rotation or tilt, which can significantly alter the morphology of the pelvic inlet, mid-pelvis, and outlet, thereby impacting the bony birth canal. The pelvic adaptations in AIS are complex and three-dimensional, involving changes in pelvic incidence, sacral slope, and pelvic tilt, which are key spinopelvic parameters influencing pelvic shape and orientation. Studies have demonstrated that pelvic incidence, a morphological parameter traditionally considered fixed, actually increases during adolescence, peaking around Risser stage 1, and correlates with changes in pelvic morphology such as pelvic height, width, and sacral dimensions [61]. These changes suggest that the pelvis in AIS patients is dynamically adapting during growth, which may influence the spatial configuration of the birth canal.

Pelvic rotation and obliquity are common in AIS and have been shown to affect spinal and pelvic measurements. For example, pelvic axial rotation does not consistently correlate with the direction of the major scoliotic curve, indicating that pelvic rotation may not reliably predict curve laterality but still contributes to the overall three-dimensional structural spine disorder [62]. Moreover, pelvic obliquity, especially in thoracolumbar/lumbar curves, is associated with postoperative coronal decompensation and residual lumbar curve progression, highlighting the clinical importance of pelvic alignment in AIS management [63]. These pelvic adaptations can alter the dimensions and orientation of the pelvic inlet and outlet, potentially affecting the bony birth canal’s adequacy for vaginal delivery.

In the context of pregnancy and delivery, particularly in the late gestational period, careful evaluation of pelvic anatomy is crucial for AIS patients, especially those with severe thoracolumbar or lumbar curves or those who have undergone spinal-pelvic fusion surgery. The altered pelvic morphology and potential fixation from fusion can lead to bony birth canal abnormalities, which may complicate vaginal delivery. Traditional two-dimensional radiographs may not sufficiently capture the complex three-dimensional pelvic deformities in these patients. Therefore, advanced imaging modalities such as three-dimensional CT reconstruction and MRI have been advocated for precise prepartum assessment of pelvic morphology [64]. These imaging techniques allow detailed visualization of the pelvic inlet, mid-pelvis, and outlet, enabling clinicians to objectively evaluate the adequacy of the bony birth canal and to plan the mode of delivery accordingly.

The use of three-dimensional imaging is particularly valuable in AIS patients with prior spinal-pelvic fusion, where the fusion mass may restrict pelvic mobility and alter pelvic dimensions. Accurate assessment of pelvic morphology can inform obstetricians about potential cephalopelvic disproportion or mechanical obstruction risks during labor. Moreover, understanding the pelvic adaptations in AIS can guide multidisciplinary management involving orthopedic surgeons, obstetricians, and anesthesiologists to optimize maternal and fetal outcomes.

In addition to bony changes, AIS-related pelvic adaptations may influence pelvic floor function. Studies have shown that AIS negatively affects pelvic floor muscle activity and function, leading to urinary dysfunction, which may further complicate pregnancy and delivery [65]. This underscores the importance of comprehensive pelvic assessment, including both bony and soft tissue structures, in the long-term management of AIS patients contemplating pregnancy.

Furthermore, spinopelvic parameters such as pelvic incidence, sacral slope, and pelvic tilt are interrelated with lumbar lordosis and thoracic kyphosis, forming a compensatory mechanism to maintain sagittal balance. Alterations in these parameters in AIS patients can influence pelvic orientation and, consequently, the shape and dimensions of the birth canal [66,67]. For example, lower pelvic incidence and sacral slope have been associated with curve progression and bracing success, indicating that pelvic morphology reflects spinal adaptability and skeletal maturity [66]. These parameters should be considered when evaluating pelvic adequacy for delivery.

In summary, AIS is commonly accompanied by pelvic rotation and tilt that alter the morphology of the pelvic inlet, mid-pelvis, and outlet, potentially impacting the bony birth canal. Late pregnancy in AIS patients, especially those with severe thoracolumbar/lumbar curves or prior spinal-pelvic fusion, necessitates meticulous pelvic anatomical evaluation. Advanced imaging modalities such as three-dimensional CT reconstruction and MRI provide precise assessment of pelvic morphology, offering objective data to guide delivery mode decisions. Additionally, the interplay between spinopelvic parameters and pelvic morphology underscores the need for a comprehensive, multidisciplinary approach to the long-term management and obstetric care of AIS patients.

## 7. Clinical Management and Pain Control in Pregnant Patients with AIS

### 7.1. Assessment and Intervention of Low Back Pain During Pregnancy

Pregnant women with AIS frequently report a higher prevalence and severity of low back pain compared to the general pregnant population, with pain often localized around the apex of the scoliotic curve or the lumbar spine. This increased incidence is attributable to the compounded biomechanical and hormonal changes during pregnancy that exacerbate pre-existing spinal deformities. The physiological adaptations of pregnancy, including increased lumbar lordosis, ligamentous laxity due to elevated relaxin and progesterone levels, and altered gait mechanics, impose additional mechanical stress on the lumbar spine and pelvis, thereby intensifying discomfort in AIS patients [60]. Moreover, the altered postural control and balance deficits observed in pregnant women with lumbopelvic pain further contribute to functional impairment and pain severity [68].

Management of pregnancy-related low back pain in AIS patients prioritizes non-pharmacological interventions to minimize fetal risk while effectively alleviating symptoms. Physical therapy forms the cornerstone of treatment, encompassing tailored exercise regimens such as lumbar stabilization and stretching exercises, which have demonstrated significant reductions in pain intensity and improvements in postural stability and trunk muscle activation in pregnant women [69]. Additionally, specialized physiotherapeutic approaches like the Schroth method, when appropriately modified for pregnancy, offer safe and targeted correction of spinal alignment and muscular imbalances, potentially mitigating curve progression and associated pain [70]. Water-based exercises and postural adjustments also provide low-impact modalities that reduce mechanical loading and enhance mobility [70].

Supportive devices, including pelvic belts and lumbar support bands, serve as adjuncts to physical therapy by stabilizing the pelvis and lumbar region, thereby reducing pain and disability. Although meta-analyses indicate only modest pain relief and minimal disability improvement with pelvic belt use during pregnancy, their low cost and ease of application justify their consideration in comprehensive management plans [71]. Kinesio taping has emerged as another promising intervention, with systematic reviews and meta-analyses confirming its efficacy in reducing low back pain and functional disability during pregnancy, particularly when applied for durations exceeding one week [72].

Non-pharmacological modalities such as transcutaneous electrical nerve stimulation (TENS), music therapy, hypnobirthing, and virtual reality-assisted physiotherapy have shown beneficial effects in alleviating pregnancy-related low back pain. Network meta-analyses rank music-relaxation therapies highest for pain reduction, while manipulation-acupuncture combinations excel in improving physical function [73]. TENS and progressive muscle relaxation exercises have demonstrated statistically significant pain relief compared to typical care [74]. The integration of virtual reality with standard physiotherapy is an innovative approach under investigation, aiming to enhance pain perception and functional outcomes [75]. Complementary therapies such as chiropractic care and osteopathic manipulative treatment (OMT) have also been reported to provide safe and effective relief from musculoskeletal pain during pregnancy, with OMT being successfully integrated into routine prenatal visits to address low back discomfort [76,77,78].

Pharmacological treatment during pregnancy requires extreme caution due to potential teratogenicity and fetal risks. Acetaminophen remains the first-line analgesic for managing low back pain in pregnant women, given its established safety profile [74]. Nonsteroidal anti-inflammatory drugs are generally avoided during the first and third trimesters due to risks of fetal complications such as premature closure of the ductus arteriosus and impaired renal function [74]. When pharmacotherapy is necessary, it should be judiciously prescribed at the lowest effective dose and for the shortest duration possible, ideally in conjunction with non-pharmacological strategies.

Patient education and health counseling are integral components of low back pain management in pregnancy, enhancing adherence to therapeutic exercises and promoting lifestyle modifications that mitigate pain and disability. Systematized narrative reviews emphasize the importance of incorporating biopsychosocial models into educational programs, addressing not only biomechanical factors but also psychosocial stressors and sleep hygiene to optimize outcomes [79]. Combining exercise with health education has been shown to yield superior reductions in pain and disability compared to education alone [80].

Emerging evidence supports the role of pelvic floor muscle training as an adjunct to physical therapy in reducing pregnancy-related low back pain, highlighting the interconnectedness of pelvic floor dysfunction and lumbar pain [81]. Furthermore, interventions such as Pilates and standardized stretching postural programs have demonstrated moderate-quality evidence for pain reduction and quality of life improvement in pregnant women suffering from low back pain [82,83].

In summary, the assessment and management of low back pain during pregnancy in AIS patients necessitate a multidisciplinary, individualized approach emphasizing non-pharmacological interventions tailored to the unique biomechanical and physiological changes of pregnancy. Physical therapy modalities, supportive devices, patient education, and cautious pharmacological use collectively contribute to effective symptom control, functional improvement, and enhanced quality of life for this vulnerable population. Continued research is warranted to establish standardized protocols and evaluate long-term outcomes of these interventions in AIS patients during pregnancy.

### 7.2. Multidisciplinary Team Collaborative Management Model

- Ideal pregnancy management requires close cooperation among obstetricians, spinal surgeons, anesthesiologists, rehabilitation therapists, and midwives.

The management of pregnancy in adult patients with idiopathic scoliosis, particularly those with a history of spinal fusion or significant spinal asymmetry, necessitates a comprehensive multidisciplinary approach to optimize maternal and fetal outcomes. Obstetricians play a central role in monitoring pregnancy progression and planning delivery, while spinal surgeons provide expertise regarding the structural and functional status of the spine, potential risks related to asymmetry progression, and implications of prior surgical interventions. Anesthesiologists contribute critical input on anesthesia planning, especially considering altered spinal anatomy that may complicate neuraxial anesthesia techniques. Rehabilitation therapists are essential for maintaining maternal mobility, managing pain, and optimizing physical function throughout pregnancy. Midwives offer continuous support during labor and delivery, ensuring patient-centered care. The integration of these specialties facilitates individualized care plans that address the complex interplay between scoliosis-related biomechanical changes and pregnancy physiology. For example, the presence of spinal instrumentation or fusion may influence anesthetic options and delivery mode decisions, requiring pre-delivery consultations among the team members. Moreover, the multidisciplinary collaboration enhances the anticipation and management of potential complications such as increased back pain, respiratory compromise, or neurological symptoms that may arise or worsen during pregnancy. This collaborative model aligns with evidence from spine care settings where multidisciplinary teams have improved surgical outcomes and reduced complications, underscoring the importance of coordinated care in complex spinal deformities [84,85,86]. In summary, the ideal pregnancy management for adult idiopathic scoliosis patients is a tightly coordinated effort among obstetricians, spinal surgeons, anesthesiologists, rehabilitation therapists, and midwives to ensure safe pregnancy progression and delivery.

- Development of individualized prenatal examination plans, pain management protocols, delivery plans, and emergency response strategies.

Given the heterogeneity of scoliosis presentations and the unique challenges posed by spinal deformities during pregnancy, individualized care plans are paramount. Prenatal examination schedules should be tailored to monitor not only fetal development but also maternal spinal status, including assessment of curve progression, pain levels, and functional capacity. Imaging modalities with minimal fetal risk, such as low-dose radiography or MRI when indicated, can be judiciously employed to evaluate spinal alignment and detect any complications. Pain management protocols must balance effective analgesia with fetal safety, often requiring multimodal approaches including physical therapy, pharmacologic agents with established safety profiles, and psychosocial support. Delivery planning involves detailed discussions regarding the mode of delivery, considering factors such as spinal fusion levels, pelvic anatomy, and obstetric indications. While vaginal delivery is often feasible, cesarean section rates may be higher in this population due to concerns about spinal instrumentation or asymmetry-related pelvic constraints [87]. Emergency response plans should be established to address potential complications such as acute neurological deterioration, severe pain crises, or anesthetic challenges during labor. These plans include rapid access to spinal surgeons and anesthesiologists familiar with the patient’s spinal anatomy and prior interventions. The use of three-dimensional printing and digital modeling technologies has been explored in complex spinal deformities to aid in surgical planning and may also assist in prenatal planning by providing detailed anatomical visualization [88]. Furthermore, enhanced recovery after surgery protocols, which emphasize multidisciplinary coordination and standardized care pathways, have demonstrated benefits in scoliosis surgery and may inform peri-delivery management strategies to optimize recovery and reduce hospital stay [86,89]. Overall, individualized prenatal and delivery plans developed through multidisciplinary collaboration are essential to address the complex needs of pregnant scoliosis patients effectively.

- Strengthening patient education to alleviate anxiety about pregnancy exacerbating scoliosis, fetal impact, and delivery safety.

Psychological distress and anxiety are common among adult scoliosis patients contemplating pregnancy, often centered on fears of curve progression during pregnancy, potential adverse effects on the fetus, and concerns regarding safe delivery. Strengthening patient education through multidisciplinary teams can significantly mitigate these anxieties by providing evidence-based information and personalized counseling. Studies have shown that psychosocial interventions involving frequent patient-provider interactions improve compliance and mental health outcomes in scoliosis patients, highlighting the importance of holistic care [90]. Education should encompass the natural history of scoliosis in pregnancy, emphasizing that while some patients may experience transient increases in back pain or minor curve changes, significant progression is uncommon. Additionally, reassurance regarding fetal safety and the availability of tailored delivery options can empower patients to make informed decisions. Multidisciplinary teams, including mental health professionals, can address emotional and psychosocial needs, facilitating coping strategies and reducing stress. The involvement of anesthesiologists in pre-delivery counseling can clarify anesthesia options and dispel misconceptions about spinal instrumentation precluding epidural or spinal anesthesia [87]. Furthermore, patient education should include discussion of rehabilitation strategies to maintain function and manage pain during pregnancy and postpartum. By fostering open communication and providing comprehensive education, multidisciplinary teams can alleviate patient fears, enhance satisfaction, and promote positive pregnancy experiences in women with idiopathic scoliosis.

## 8. Selection of Delivery Methods and Considerations for Perinatal Anesthesia

### 8.1. Decision Factors for Vaginal Delivery vs. Cesarean Section

The decision-making process regarding the mode of delivery in pregnant women with AIS or adult idiopathic scoliosis is multifactorial and must be individualized, taking into account the severity of spinal curvature, pelvic anatomy, prior spinal surgeries, fetal status, and maternal preferences. Most women with AIS can successfully achieve vaginal delivery without significant complications. This is supported by evidence indicating that the overall cesarean section rate in scoliosis patients is comparable to that of the general population, suggesting that scoliosis alone is not an absolute indication for cesarean delivery [91,92]. The severity of the spinal asymmetry plays a crucial role; mild to moderate curves generally do not impede the mechanics of labor or delivery, whereas severe deformities, particularly those involving the thoracic cage or pelvis, may pose challenges.

Pelvic assessment is essential in the obstetric evaluation of these patients. Severe scoliosis can alter pelvic morphology and reduce pelvic dimensions, potentially complicating the passage of the fetus through the birth canal. In cases where pelvic inlet or outlet dimensions are compromised, vaginal delivery may be difficult or unsafe. Radiological and clinical pelvic evaluations help determine whether vaginal delivery is feasible. Additionally, the presence of significant thoracic cage deformities may restrict respiratory function and limit the mother’s ability to perform effective expulsive efforts during labor, which could necessitate cesarean delivery [93,94].

A history of extensive spinal fusion surgery, especially long-segment fusion extending to the lower lumbar vertebrae (L5) or sacrum (S1), is a critical factor influencing delivery mode. Such surgeries can reduce sacral mobility and pelvic flexibility, which are vital for the physiological adaptations during vaginal delivery. Consequently, elective cesarean section is often recommended for women with prior long-segment spinal fusion to avoid labor dystocia and potential maternal or fetal complications [91,92]. However, the decision must be balanced against the risks associated with cesarean delivery and anesthesia in this population, which can be complicated by spinal deformities and prior instrumentation.

Fetal considerations, including fetal size, presentation, and well-being, also influence delivery planning. In some cases, fetal malposition or distress may necessitate cesarean delivery regardless of maternal spinal status. Maternal preference and informed consent are paramount; women should be counseled thoroughly about the risks and benefits of each delivery mode in the context of their spinal condition and obstetric factors [91].

Severe thoracic deformities or significant trunk imbalance may also impact the choice of delivery mode. These deformities can limit the mother’s ability to assume optimal labor positions or perform effective pushing, thereby increasing the likelihood of cesarean section. In such scenarios, a multidisciplinary team approach involving obstetricians, anesthesiologists, and spine specialists is essential to optimize maternal and fetal outcomes [95].

Anesthetic considerations further complicate delivery planning. Patients with scoliosis, especially those with prior spinal surgery, may present challenges for neuraxial anesthesia due to altered spinal anatomy and scarring. Failed or difficult spinal or epidural anesthesia has been reported more frequently in this population, sometimes necessitating general anesthesia for cesarean delivery [91,96]. This risk may influence the decision toward elective cesarean section in some cases to ensure controlled anesthesia management.

In summary, while most women with AIS can undergo vaginal delivery successfully, the decision between vaginal birth and cesarean section must be individualized. Key factors include the severity of scoliosis, pelvic anatomy, history of spinal fusion surgery (particularly long-segment fusion to L5 or S1), presence of severe thoracic asymmetry, fetal status, and maternal preference. Elective cesarean section is often recommended for patients with extensive spinal fusion to the pelvis or severe deformities that may impair labor mechanics or respiratory function. A multidisciplinary approach is essential to optimize outcomes, with careful pre-delivery planning and counseling to address the unique challenges posed by scoliosis in pregnancy and delivery.

### 8.2. Anesthetic Risk Assessment and Strategies

In the management of pregnant patients with AIS, anesthetic risk assessment and strategy formulation are critical due to the unique challenges posed by spinal deformities and prior surgical interventions. Regional anesthesia, particularly neuraxial techniques such as epidural or spinal anesthesia, is generally preferred for labor analgesia and cesarean delivery because it provides effective pain control while minimizing fetal exposure to systemic anesthetics. However, AIS can complicate the administration of neuraxial anesthesia by increasing the technical difficulty and failure rates of needle placement. The spinal rotation, presence of surgical scars, and fused vertebral segments alter the usual anatomical landmarks, making identification of the epidural or subarachnoid space challenging. Studies have shown that patients with AIS who have undergone spinal fusion may require more attempts and experienced anesthesiologists to successfully place epidural catheters, with some reports indicating a lower success rate of neuraxial anesthesia compared to healthy controls [59,97]. This necessitates careful preoperative planning and consideration of adjunctive imaging techniques such as ultrasound or fluoroscopy to guide needle placement. Ultrasound imaging has been increasingly utilized to visualize the spinal anatomy, identify intervertebral spaces, and estimate needle insertion depth, thereby improving the success rate and safety of neuraxial blocks in patients with distorted spinal anatomy [98,99]. In cases where regional anesthesia is contraindicated or unsuccessful, a well-prepared general anesthesia plan must be in place to manage potential airway difficulties and respiratory compromise, especially in patients with severe scoliosis or associated neuromuscular conditions. The anesthetic approach should also consider the potential for intraoperative complications such as malignant hyperthermia, particularly in patients with underlying neuromuscular disorders [100,101]. Multidisciplinary collaboration among anesthesiologists, obstetricians, and orthopedic surgeons is essential to optimize maternal and fetal outcomes. Furthermore, contingency plans including readiness for emergency cesarean section under general anesthesia should be established. Overall, the anesthetic management of pregnant AIS patients requires individualized risk assessment, utilization of advanced imaging guidance for neuraxial anesthesia, and preparedness for alternative anesthetic techniques to ensure safety and efficacy [6,102,103].

## 9. Postpartum Recovery, Progression of Scoliosis, and Impact on Offspring

### 9.1. Postpartum Spinal Status and Functional Recovery

The postpartum period in women with idiopathic scoliosis presents a complex interplay of physiological and biomechanical changes that significantly influence spinal status and functional recovery. After delivery, the hormonal milieu undergoes a rapid shift, with a marked decline in pregnancy-associated hormones such as relaxin and progesterone. This hormonal regression facilitates the restoration of ligamentous tension and joint stability, which were previously relaxed to accommodate fetal growth and prepare for childbirth. However, despite this hormonal normalization, the biomechanical alterations acquired during pregnancy—such as increased body weight, altered center of gravity, and changes in muscle mass and strength—may persist and continue to impact spinal mechanics adversely. These persistent changes can exacerbate pre-existing spinal deformities or contribute to new biomechanical stresses, potentially influencing the progression or symptomatology of scoliosis in the postpartum period [6].

Given these challenges, early initiation of safe and targeted postpartum rehabilitation is critical. Rehabilitation programs should prioritize the restoration of core muscle strength, which plays a pivotal role in maintaining spinal alignment and stability. Strengthening the deep abdominal and paraspinal musculature can help counteract the mechanical disadvantages imposed by scoliosis and pregnancy-related musculoskeletal adaptations. Additionally, postural correction exercises are essential to address compensatory postural deviations that may have developed during pregnancy. Such interventions not only alleviate chronic back pain—a common complaint among postpartum women with scoliosis—but also prevent functional decline and improve overall quality of life. The timing of rehabilitation is crucial; commencing these interventions as early as medically feasible postpartum can optimize outcomes by leveraging the plasticity of musculoskeletal tissues and preventing maladaptive compensatory patterns [6].

Radiological assessment remains a cornerstone in monitoring scoliosis progression and spinal status after pregnancy. It is recommended that standing full-spine radiographs be obtained between six to twelve months postpartum to evaluate any significant changes in the Cobb angle compared to pre-pregnancy baselines. This interval allows for the resolution of transient pregnancy-related postural changes and provides a more accurate depiction of the structural spinal status. Studies have demonstrated that some patients experience an increase in scoliosis curvature postpartum, with a subset showing a Cobb angle progression of 3° or more, which may necessitate clinical intervention or modification of rehabilitation strategies [6]. Regular imaging follow-up facilitates timely identification of progression, enabling clinicians to tailor management plans accordingly and potentially mitigate long-term functional impairment.

In summary, the postpartum recovery of spinal function in women with idiopathic scoliosis is influenced by the interplay of hormonal normalization, persistent biomechanical alterations, and the adequacy of rehabilitation efforts. Early, focused rehabilitation targeting core strength and posture correction is essential to counteract the residual effects of pregnancy on spinal mechanics and to prevent chronic pain and functional decline. Concurrently, systematic radiographic monitoring is indispensable for detecting scoliosis progression and guiding clinical decision-making. Multidisciplinary care involving obstetricians, orthopedic specialists, physiotherapists, and radiologists is paramount to optimize postpartum spinal health and functional recovery in this patient population.

### 9.2. Genetic Predisposition of AIS and Offspring Monitoring

AIS is widely recognized as a multifactorial disorder with a significant genetic component, exhibiting familial aggregation and polygenic inheritance patterns. Multiple studies have demonstrated that children of AIS patients possess a higher risk of developing the condition compared to the general population, underscoring the importance of genetic predisposition in AIS pathogenesis. For instance, a large population-based study involving over 600,000 adolescents revealed that those with one or both parents affected by spinal deformities had significantly increased odds ratios (ORs) for adolescent spinal deformities, including AIS, with ORs of 1.46, 1.74, and 2.58 for paternal, maternal, and both parents affected, respectively [104]. This familial clustering supports the hypothesis that genetic factors contribute substantially to AIS susceptibility.

Genetic studies have identified numerous candidate genes and loci associated with AIS risk, reflecting its polygenic nature. Variants in genes such as SOX9, TBX1, UTS2R, POC5, and ADAMTSL2 have been implicated in AIS predisposition across diverse populations. For example, SOX9 gene variants, including those in enhancer regions, have been associated with AIS susceptibility in Northwest Indian populations, suggesting that regulatory elements influencing gene expression may play a critical role in disease development [105]. Similarly, the TBX1 gene variant rs1978060 was found to be functionally associated with AIS in the Chinese Han population, with decreased TBX1 expression correlating with curve severity, indicating a potential role in disease progression [106]. Rare mutations in UTS2R were also linked to AIS susceptibility, with increased expression of UTS2R observed in AIS patients, highlighting the involvement of urotensin II signaling pathways [107]. Furthermore, variants in POC5, a ciliary gene, were prevalent in French-Canadian and British AIS cohorts, suggesting ciliary dysfunction as a pathogenic mechanism [108]. The aberrant interaction between mutated ADAMTSL2 and LTBP4, which upregulates TGF-βsignaling, has also been implicated in AIS pathogenesis, indicating that extracellular matrix and signaling pathway disruptions contribute to the disorder [109].

Given the genetic predisposition and the increased risk in offspring, regular monitoring of children born to AIS patients is crucial for early detection and intervention. Screening methods such as the Adams forward-bend test, which assesses spinal asymmetry and rib hump, combined with scoliometer measurements of trunk rotation, are practical and non-invasive tools for initial scoliosis screening during the rapid growth phase of adolescence, particularly between ages 10 and 14 when the risk of curve progression is highest [110]. Early identification through such screening allows timely referral for radiographic evaluation and initiation of conservative treatments like bracing, which have been shown to effectively halt or slow curve progression.

In addition to physical screening, genetic counseling plays a vital role in managing familial AIS risk. Counseling should provide clear information about the hereditary nature of AIS, emphasizing that while genetic factors increase susceptibility, the condition is multifactorial and not solely determined by genetics. This approach helps to inform families about the elevated risk to offspring without causing undue anxiety. Genetic counseling also underscores the importance of early screening and intervention, which can improve long-term outcomes and quality of life. It is essential to balance risk communication with reassurance, highlighting that many individuals with genetic predisposition do not develop severe scoliosis and that effective management strategies exist.

Moreover, recent research suggests that AIS shares genetic overlaps with other complex conditions, such as schizophrenia, indicating common pathways related to neuronal development and cellular signaling [111]. This insight may eventually refine risk assessment and monitoring strategies by integrating broader genetic and biological markers. However, the heterogeneity of AIS genetics and the incomplete understanding of gene-environment interactions necessitate ongoing research and cautious interpretation of genetic risk.

In summary, AIS exhibits a clear genetic predisposition with familial aggregation and polygenic inheritance. Offspring of AIS patients have a significantly higher risk of developing scoliosis, warranting regular spinal screening during critical growth periods using clinical methods like the Adams forward-bend test. Genetic counseling should be offered to affected families to communicate risks effectively, promote early detection, and alleviate unnecessary anxiety. Continued advances in genetic research will enhance understanding of AIS pathogenesis and improve strategies for offspring monitoring and management.

## 10. Future Research Directions and Comprehensive Management Outlook

### 10.1. Development of Clinical Guidelines Based on Evidence-Based Medicine

The development of clinical guidelines for the management of adult patients with AIS, particularly during pregnancy, remains an unmet need in current medical practice. Despite the prevalence of AIS and the unique challenges posed by pregnancy-related physiological changes, there is a conspicuous absence of authoritative, evidence-based clinical guidelines tailored specifically to this population. This gap underscores the urgent necessity for guidelines grounded in robust, large-scale prospective cohort studies or registry data that can provide high-quality evidence to inform clinical decision-making. Existing research on AIS has predominantly focused on adolescent populations, with limited extrapolation to adult patients, especially those undergoing pregnancy. For example, recent efforts in adolescent scoliosis have emphasized evidence-based personalized approaches that integrate clinical trial data and patient-centered care to optimize outcomes and treatment adherence [112]. However, these approaches have yet to be adapted or validated for adult patients with AIS, whose spinal biomechanics and comorbidities differ significantly from adolescents.

Moreover, the current literature lacks comprehensive quantification of the true risk factors for scoliosis progression during pregnancy, as well as the specific impacts of pregnancy on pain and functional status in this patient group. Understanding these parameters is critical for developing guidelines that can effectively balance maternal and fetal health considerations with spinal asymmetry management. The complexity of scoliosis as a three-dimensional spinal asymmetry, coupled with the biomechanical and hormonal changes during pregnancy, necessitates a nuanced approach to risk stratification and therapeutic interventions [113]. Existing rehabilitation guidelines for AIS, such as those recently proposed by the Chinese Society of Physical Medicine and Rehabilitation, provide a framework for adolescent patients but do not address pregnancy-related issues or adult-specific concerns [113]. This highlights the need for multidisciplinary collaboration involving orthopedic surgeons, rehabilitation specialists, obstetricians, and evidence-based medicine experts to develop comprehensive, pregnancy-specific clinical guidelines.

The readiness for implementing evidence-based practices in scoliosis rehabilitation has been assessed in adolescent populations, revealing a generally positive attitude among healthcare providers but also identifying barriers related to organizational environment and knowledge gaps [114]. Translating this readiness into effective guideline development for adult patients with AIS during pregnancy will require targeted education and resource allocation to ensure that clinicians are equipped to apply evidence-based recommendations in complex clinical scenarios. Furthermore, the historical evolution of scoliosis treatment demonstrates a pendulum swing between conservative and surgical approaches, with recent advances in brace technology and rehabilitation emphasizing the importance of evidence-based conservative management [115]. This historical context supports the integration of conservative strategies within clinical guidelines, particularly for pregnant patients where surgical options may be limited or contraindicated.

In summary, the development of clinical guidelines for adult patients with AIS during pregnancy must be predicated on high-quality evidence derived from large prospective cohorts or registries that elucidate the natural history and risk factors of scoliosis progression in this unique population. These guidelines should quantify the impact of pregnancy on pain and functional outcomes, incorporate multidisciplinary expertise, and leverage the growing readiness among healthcare providers to adopt evidence-based practices. Only through such rigorous, evidence-based guideline development can clinicians be empowered to deliver personalized, safe, and effective care to adult patients with AIS navigating the challenges of pregnancy.

### 10.2. Emerging Technologies in Evaluation and Management

The integration of emerging technologies into the evaluation and management of adult idiopathic scoliosis, particularly in the context of pregnancy, represents a transformative advancement in clinical practice. Wearable sensors and dynamic biomechanical analysis have become invaluable tools for objectively assessing spinal load and postural control during pregnancy and daily activities. These devices enable continuous monitoring of spinal biomechanics in real-world settings, capturing data on spinal alignment, movement patterns, and load distribution that traditional static imaging cannot provide. For pregnant patients with adult idiopathic scoliosis, wearable technology facilitates the quantification of spinal stress and compensatory postural adjustments that occur due to physiological changes, such as increased lumbar lordosis and anterior weight shift. This objective data supports personalized management strategies aimed at minimizing discomfort and preventing curve progression during pregnancy. For example, inertial measurement units and pressure sensors embedded in wearable systems can detect subtle deviations in posture and gait, allowing clinicians to tailor physiotherapeutic interventions and monitor their effectiveness over time [116].

Artificial intelligence (AI)-assisted imaging analysis is another frontier enhancing the precision of scoliosis evaluation and prognostication. Traditional radiographic assessments, such as the Cobb angle measured on two-dimensional X-rays, have limitations in capturing the three-dimensional complexity of spinal deformities. Recent developments in AI, particularly deep learning algorithms, have enabled automated, accurate, and reproducible measurements of spinal curvature from various imaging modalities. For instance, convolutional neural networks have been trained to synthesize radiograph-comparable images from simple RGB photographs, providing radiation-free, rapid assessments of scoliosis severity and curve classification with high diagnostic accuracy [117]. Moreover, AI models can integrate longitudinal imaging data and clinical parameters to predict curve progression risk in adulthood and during pregnancy, facilitating early intervention and individualized treatment planning. The use of three-dimensional imaging techniques combined with AI segmentation algorithms allows for comprehensive evaluation of spinal alignment, vertebral rotation, and structural changes in intervertebral discs and ligaments, which are critical for understanding the biomechanical impact of pregnancy on scoliosis [38,118]. These advancements promise to refine risk stratification and optimize timing for therapeutic interventions.

Telemedicine and digital rehabilitation platforms have emerged as essential components in the long-term management of adult idiopathic scoliosis patients, especially those navigating pregnancy-related challenges. Remote monitoring technologies enable continuous patient engagement, adherence tracking, and timely adjustments to treatment regimens without the need for frequent in-person visits. Telehealth visits have been validated for scoliosis assessment, demonstrating high agreement with in-person clinical measurements of trunk rotation and postural asymmetry, thus ensuring reliable remote evaluation [119]. Digital platforms can deliver tailored physiotherapy programs, including physiotherapy specific scoliosis exercises, with real-time feedback and progress tracking, enhancing patient compliance and outcomes [120]. Furthermore, integration of wearable sensor data into telemedicine systems allows clinicians to monitor biomechanical parameters continuously, enabling proactive management of spinal load and posture during pregnancy. This approach is particularly beneficial for pregnant patients who may face mobility limitations or increased healthcare access barriers. The convenience and accessibility of digital rehabilitation foster multidisciplinary collaboration and patient-centered care, which are crucial for addressing the complex needs of adult scoliosis patients during pregnancy.

Collectively, these emerging technologies—wearable sensors, AI-assisted imaging, and telemedicine platforms—are revolutionizing the evaluation and management of adult idiopathic scoliosis in the context of pregnancy. They provide objective, precise, and continuous data that inform personalized treatment strategies, improve monitoring of disease progression, and enhance patient engagement. Future research should focus on integrating these technologies into standardized care pathways, validating their efficacy in diverse populations, and exploring their potential to predict and mitigate pregnancy-related scoliosis complications. The convergence of biomechanical monitoring, AI analytics, and digital health promises to elevate the quality and accessibility of scoliosis care for pregnant patients, ultimately improving maternal and fetal outcomes.

### 10.3. Construction of a Lifelong Health Management Model

The construction of a comprehensive lifelong health management model for adult patients with idiopathic scoliosis, particularly those transitioning from adolescence to adulthood and facing pregnancy-related challenges, is imperative to optimize long-term outcomes and quality of life. This model advocates for a seamless continuum of care that begins in adolescence and extends through adulthood, incorporating integrated services such as fertility counseling, pregnancy management, and postpartum rehabilitation. Evidence from long-term follow-up studies of AIS patients underscores the necessity of continuous monitoring and intervention to address curve progression, pain, and functional limitations that may persist or evolve over decades [121]. The transition from pediatric to adult care is a critical juncture where discontinuities often occur, potentially leading to suboptimal management of spinal deformities and associated comorbidities. Establishing structured pathways that facilitate smooth handovers between pediatric and adult healthcare providers ensures that patients receive consistent surveillance and timely interventions tailored to their evolving needs.

Incorporating reproductive health into this model is essential, given the unique concerns of female patients with scoliosis regarding fertility, pregnancy, and childbirth. Current literature reveals that while most women with a history of AIS or spinal fusion can conceive and deliver without significant perinatal complications, they may experience higher rates of cesarean sections and pregnancy-related back pain, especially when fusion extends to lower lumbar vertebrae [91]. Therefore, integrating fertility counseling and pregnancy-specific management into the care continuum can empower patients with informed decision-making and personalized care plans. Postpartum rehabilitation focusing on musculoskeletal recovery and pain management further supports functional restoration and quality of life.

Patient education is a cornerstone of this lifelong management approach. Enhancing patients’ self-management capabilities through targeted education programs improves adherence to conservative treatments such as bracing and physiotherapeutic scoliosis-specific exercises, which have demonstrated efficacy in stabilizing curves and improving quality of life [122,123]. Moreover, addressing psychological health is vital, as mental health disorders can adversely affect postoperative pain management and recovery trajectories [124]. Clinician-led mental health discussions and counseling have been shown to positively influence clinical outcomes, underscoring the need for psychosocial support integrated within scoliosis care [125].

To advance this model, fostering multicenter collaborative research is crucial for accumulating high-quality evidence on the long-term prognosis and management strategies tailored to this special population. Current data on adult outcomes, especially concerning pregnancy and psychosocial aspects, remain limited and heterogeneous [92]. Multicenter studies can provide robust datasets to elucidate factors influencing curve progression, pain, functional status, and reproductive outcomes, thereby refining clinical guidelines and individualized care pathways. Additionally, such collaborations can facilitate the development and validation of standardized patient-reported outcome measures like the Staffordshire Questionnaire for AIS, enhancing the assessment of health-related quality of life across the lifespan [126].

In summary, constructing a lifelong health management model for adult idiopathic scoliosis patients necessitates a multidisciplinary, patient-centered approach that ensures continuity of care from adolescence through adulthood. This model integrates reproductive health services, emphasizes patient education and psychological support, and is underpinned by collaborative research efforts to generate evidence-based practices. Such a framework aims to mitigate long-term complications, optimize functional outcomes, and enhance the overall well-being of this unique patient population.

## 11. Conclusions

The long-term management of adult patients with AIS represents a complex and dynamic clinical challenge that demands a lifelong, multidisciplinary approach. From an expert perspective, this review underscores the necessity of balancing vigilant natural history monitoring, symptom control, functional preservation, and timely surgical intervention when indicated. The evolving understanding of AIS in adulthood highlights that management strategies must be individualized, taking into account the unique biomechanical and clinical nuances that arise over time, particularly during critical life events such as pregnancy.

Pregnancy emerges as a pivotal juncture in the continuum of care for female adult patients with AIS. The biomechanical alterations and physiological demands imposed by gestation introduce distinct challenges that can exacerbate symptoms, notably pain, and potentially influence spinal curvature dynamics. However, the current body of evidence suggests that while pregnancy may not significantly alter the long-term progression of the scoliotic curve, it can intensify symptomatology, necessitating proactive and anticipatory management. This nuanced understanding calls for a delicate balance between acknowledging the potential risks and avoiding unnecessary interventions that may not confer additional benefit. Based on the available evidence, the level of recommendation for routine intervention based solely on AIS status during pregnancy is weak, and risk stratification should primarily focus on pre-existing symptom severity, curve magnitude, and the presence of respiratory or neurological compromise.

A key insight from the reviewed literature is the critical role of multidisciplinary, prospective planning tailored to the individual patient’s spinal and pelvic anatomy, obstetric history, and overall health status. Such an approach facilitates informed decision-making regarding delivery methods, with cesarean section reserved for obstetric indications rather than scoliosis alone. The feasibility of regional anesthesia, despite its technical complexity in the context of spinal asymmetry, further exemplifies the importance of specialized expertise and individualized care plans. This balance between obstetric safety and spinal considerations epitomizes the integrative management philosophy necessary for optimizing outcomes.

Postpartum care and rehabilitation constitute another essential pillar in the continuum of AIS management. Systematic rehabilitation programs aimed at restoring function, alleviating pain, and enhancing quality of life are vital, as is the implementation of spinal health screening protocols for offspring, given the hereditary and developmental implications of scoliosis. These components reflect a holistic approach that extends beyond immediate clinical concerns to encompass long-term health trajectories for both mother and child.

From a research standpoint, the review highlights significant gaps in high-quality evidence guiding the management of adult patients with AIS, particularly concerning pregnancy-related outcomes and standardized care pathways. Future investigations should prioritize longitudinal, prospective studies that integrate advanced imaging, biomechanical modeling, and patient-reported outcomes to refine risk stratification and therapeutic algorithms. Specifically, research is needed to develop validated risk stratification tools that can guide the intensity of monitoring and intervention during pregnancy and postpartum. The integration of emerging technologies such as three-dimensional spinal assessment tools, wearable sensors for functional monitoring, and telemedicine platforms for remote management holds promise for enhancing personalized care and facilitating continuous monitoring.

Moreover, the development of consensus-driven, evidence-based guidelines is imperative to harmonize clinical practice and reduce variability in care delivery. These guidelines should explicitly incorporate risk stratification frameworks and provide clear levels of recommendation (e.g., strong, weak) for key management decisions across the lifespan, including during pregnancy. Such guidelines should incorporate multidisciplinary input from orthopedic surgeons, obstetricians, anesthesiologists, physiatrists, and rehabilitation specialists to address the multifaceted needs of adult patients with AIS comprehensively. Emphasizing patient education and shared decision-making will further empower individuals to actively participate in their care, fostering adherence and optimizing outcomes.

In conclusion, the management of adult patients with AIS, particularly women navigating pregnancy, demands a sophisticated, patient-centered approach that integrates current evidence with clinical expertise. A critical component of this approach is the application of clear risk stratification and evidence-based levels of recommendation to inform clinical decision-making. By balancing the diverse research perspectives and clinical findings, clinicians can tailor interventions that mitigate risks, preserve function, and enhance quality of life across the lifespan. Continued research efforts and technological advancements are essential to fill existing knowledge gaps, standardize care, and ultimately improve the health trajectories of adult patients with AIS and their families. This comprehensive, lifelong management paradigm represents the future direction for optimizing outcomes in this complex patient population.

## Data Availability

The original contributions presented in the study are included in the article. Further inquiries can be directed to the corresponding authors.
